# Identification of target genes in cardiomyopathy with fibrosis and cardiac remodeling

**DOI:** 10.1186/s12929-018-0459-8

**Published:** 2018-08-16

**Authors:** Jianquan Zhao, Tiewei Lv, Junjun Quan, Weian Zhao, Jing Song, Zhuolin Li, Han Lei, Wei Huang, Longke Ran

**Affiliations:** 1 Department of Cardiology, Bayannaoer City Hospital, 35 Xinhua District, Bayannaoer, 015000 Inner Mongolia China; 2 Department of Cardiology, Children’s hospital, Chongqing Medical University, Chongqing, China; 3 Department of Bioinformatics, Chongqing Medical University, 1 Yixueyuan Road, Yuzhong District, Chongqing, 400016 China; 4 Department of Vascular Cardiology, the First Affiliated Hospital of Chongqing, Medical University, Chongqing, China

**Keywords:** Dilated cardiomyopathy, Bioinformatics, Microarray, Heart failure

## Abstract

**Background:**

Identify genes probably associated with chronic heart failure and predict potential target genes for dilated cardiomyopathy using bioinformatics analyses.

**Methods:**

Gene expression profiles (series number GSE3585 and GSE42955) of cardiomyopathy patients and healthy controls were downloaded from the Expression Omnibus Gene (GEO) database. Differential expression of genes (DEGS) between the two groups of total 14 cardiomyopathy patients and 10 healthy controls were subsequently identified by limma package of R. Database for Annotation, Visualization, and Integrated Discovery (DAVID Tool), which is an analysis of enriched biological processes. Search Tool for the Retrieval Interacting Genes (STRING) was used as well for the analysis of protein-protein interaction network (PPI). Prediction of the potential drugs was suggested based on the preliminarily identified genes using Connectivity Map (CMap).

**Results:**

Eighty-nine DEGs were identified (57 up-regulated and 32 down-regulated). The most enrichment Gene Ontology (GO) terms (*P* < 0.05) contain genes involved in extracellular matrix (ECM) and biological adhesion signal pathways (*P* < 0.05, ES > 1.5) such as ECM-receptors, focal adhesion and transforming growth factor beta (TGF-β), etc. Fifty-one differentially expressed genes were found to encode interacting proteins. Eleven key genes along with related transcription factors were identified including CTGF, POSTN, CORIN, FIGF, etc.

**Conclusion:**

Bioinformatics-based analyses reveal the targeted genes probably associated with cardiomyopathy, which provide clues for pharmacological therapies aiming at the targets.

## Background

Patients with dilated cardiomyopathy (DCM) often presents with progressive congested heart failure, arrhythmia and thromboembolic disease in forms of left ventricular mural thrombus and/or strike [1]. DCM is seen as a major cause of heart failure apart from coronary heart disease and hypertension [2]. Heart failure is a progressive chronic disease with a 5-year-suvival rate less than 50% [3]. The histologically confirmed diagnosis indicated that prevalence of DCM is 14 to 52% among population proven previous history of myocarditis [4]. High morbidity and mortality underscore the necessity of deeper investigation of the underlying mechanism responsible for development of heart failure in DCM [5]. B-type natriuretic peptides (BNP) is a commonly used biomarker so far for diagnosis of DCM. However, the biomarkers are not so specific since the increased levels of the biomarkers sometimes indicate variety of cardiovascular diseases caused by heterogenetic etiologies, and cannot be explained by impaired left ventricular function alone [6]. The major goal for the treatment of DCM is to reduce the mortality and morbidity rate, to relief symptoms, and to prevent or, in some extent, reverse ventricular remodeling [7]. Fett et al. reported the necessity of application of polymerase chain reaction (PCR) prior to the immunosuppressive therapy [8]. Hamshere *at el* provided a therapy by administration of granulocyte colony-stimulating factor (G-CSF) with intracoronary autologous bone marrow-derived cells (BMCs) to improve left ventricular systolic function in patients with DCM [9]. Beta-blocker and pace maker inhibitor ivabradine has been proved to have positive effects in reversing cardiac remodeling. However, reactions to beta-blocker or ivabradine vary based on the cases. In some individuals the left ventricular ejection fraction is improved significantly as a result of reversed of attenuated remodeling while in the others it remains [10]. More studies are needed to focus on treatment that improves the outcome of patients with DCM, to precisely make the diagnosis of the disease based on screening of biomarkers. These studies can improve prognosis of patients by lowering the risk of development of heart failure and relevant complications. So it is crucial to understand the mechanism and find biomarkers with a good specificity and sensitivity.

Gene chip microarray database has an open access. Gene chip technique is a widely used approach in analyzing gene function in post-genome era [11]. It is a High-throughput sequencing technique with optimal specificity and sensitivity. Previous studies have partially demonstrated the mechanism underlying DCM by this approach [12, 13]. In this study, two platforms (GPL96 and GPL6244) are incorporated to analyze differential expression genes, enrichment of GO terms or pathways and protein-protein interactions in DCM and predict potential targets and drugs for a better treatment of the disease.

## Methods

### Microarray gene expression

Gene expression profiles with series number GSE3585 based on platform GPL96 and series number GSE42955 on platform GPL6244 were downloaded from the Expression Omnibus Gene (GEO) database (http://www.ncbi.nlm.nih.gov/geo/). Data of seven cardiomyopathy patients and seven healthy controls were randomly selected from each platform, respectively. The total sample of the two patient groups is 14 and the healthy controls are 10 subjects. The samples from all selected subjects had been hybridized on the Affymetrix Human Genome U133A array on a GPL96/GPL6244 platform (Affymetrix, Santa Clara, CA, USA).

### Identification of Differential Expressed Genes (DEG)

Limma of R/bio-conductor was used for screening of the DEGs (settings: *P* < 0.05, log2 (Fold Change)>/=1). Fifty-seven up-regulated (gene set A) and 32 down -regulated (gene set B) genes were identified. Hierarchical clustering and visualization were made by Heat-map package of R.

### Enrichment analysis of significant modules

The Database for Annotation, Visualization and Integrated Discovery (DAVID) [14] provides a non-line comprehensive set of functional annotation tools for biological interpretation of large gene lists. DAVID was used here to group the functions of DEGs in modules, identify enriched biological processes and cellular components, and identify the pathways associated with the DEGs in the most significant modules. Function and pathway terms were retrieved from the Gene Ontology (*p* < 0.05, Benjamin< 0.01) and the Kyoto Encyclopedia of Genes and Genomes (KEGG) (*p* < 0.05, Benjamin< 0.01) databases, respectively.

### Analysis of protein-protein interaction network

The topological properties of the PPI networks were analyzed using Network Analyzer available in Cytoscape. Search Tool for the Retrieval Interacting Genes 10.0 (http://string-db.org/) [15] provide online analysis of interactions among DEG-encoding proteins. The network of interactions was then imported into Cytoscape [16], degree ≥5 was set to filter crucial proteins in the middle of the network.

### Analysis of transcription factors

PASTAA [17] was used for predictive analysis of transcription factors of DCMs, *P* value calculated from hyper geometric distribution was used to evaluate the correlation between the DCMs and transcription factor. TRAP [18] was used to predict the correlation. Geneset C,D was uploaded to the database and JASPAR, (version 2016) [19] was used to predict the DNA binding site.

### Acquisition of target genes of drugs

Connectivity map [20] is an online implement that provides gene transcription-expression profile of thousands of genomes reflecting how cultured mammalian cell react to administration of 1309 kinds of bioactive modules in terms of gene-expression. CMap was used to identify potential unknown effect of existing drugs. Up-regulated gene set (gene set A) and down-regulated gene set (gene set B) were uploaded to CMap, EBAYES was used to calculate *P*-value, PEBAYES-value< 0.05 was considered significant, and could be a potential molecule in treatment of DCM. The half maximal inhibitory concentration (IC50) is a measure of the effectiveness of a substance in inhibiting a specific biological or biochemical function. In previous studies effectiveness of different drugs were demonstrated and published. Thus, results obtained by CMap were uploaded to NCBI PubChem database for verification.

## Results

### Identification of DCGs

**Fig. 1:**
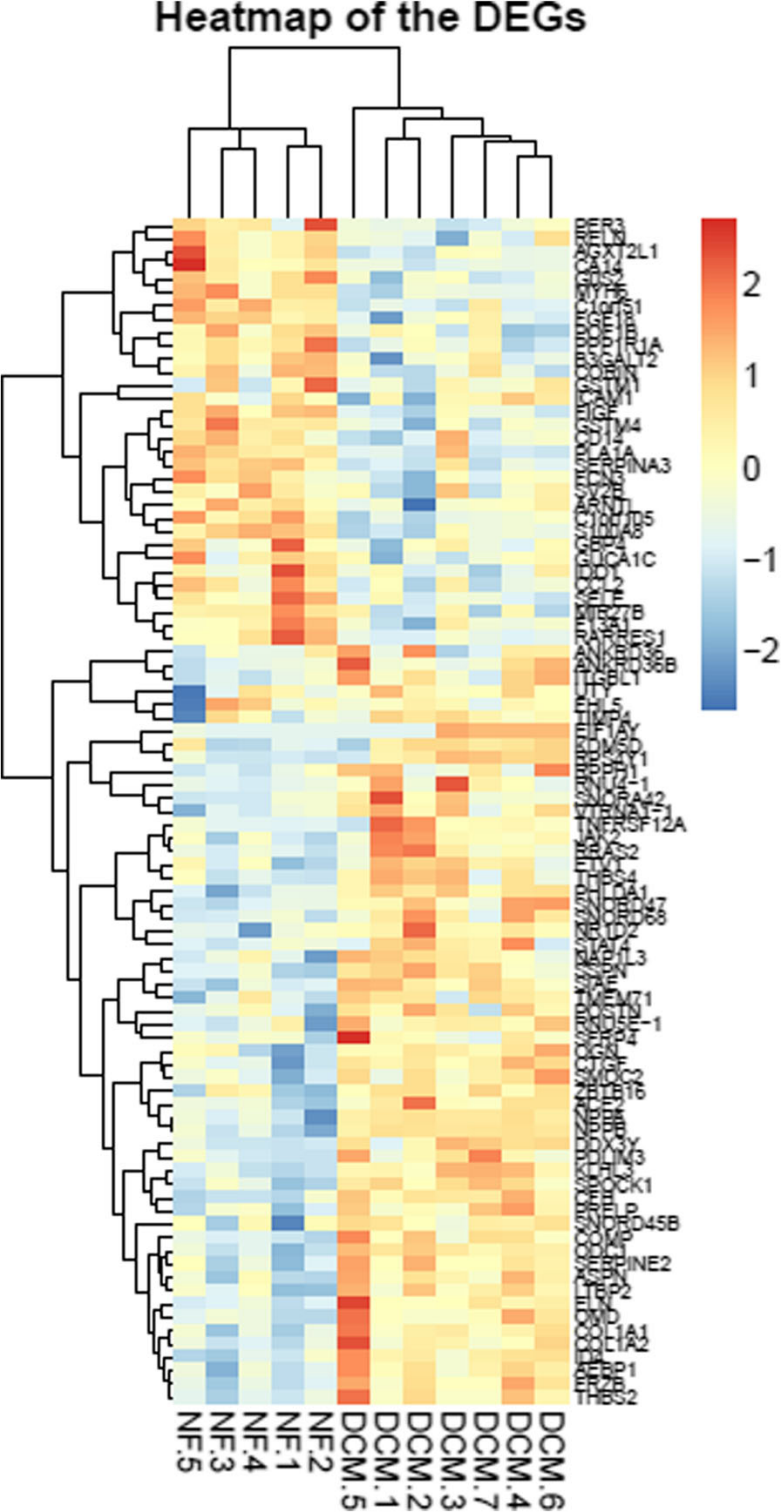
The heat-map of differential expression genes (*P* < 0.05, |logFC| > 1). NF: Control group, DCM: DCM group, up-regulated genes were in red and down-regulated genes were in blue

Compared to the control group, 89 differential expression genes (DEG) are identified in the DCM group (57 up-regulated and 32 down-regulated), Fig. 1 is the hierarchical clustering heat-map.

### Functional enrichment analysis

In GO functional and enrichment analysis, the most enriched GO terms associated with up-regulated genes are extracellular matrix concerning CC (*P* = 3.810^− 11^), extracellular region (*P* = 5 × 10^-14^) and biological adhesion (*P* = 1.5 × 10^-6^), the most enriched GO terms associated with down-regulated genes are response to injury concerning MF (*P* = 1.5 × 10^-6^) and extracellular region (*P* = 5 × 10^-14^) concerning CC.

**Fig. 2:**
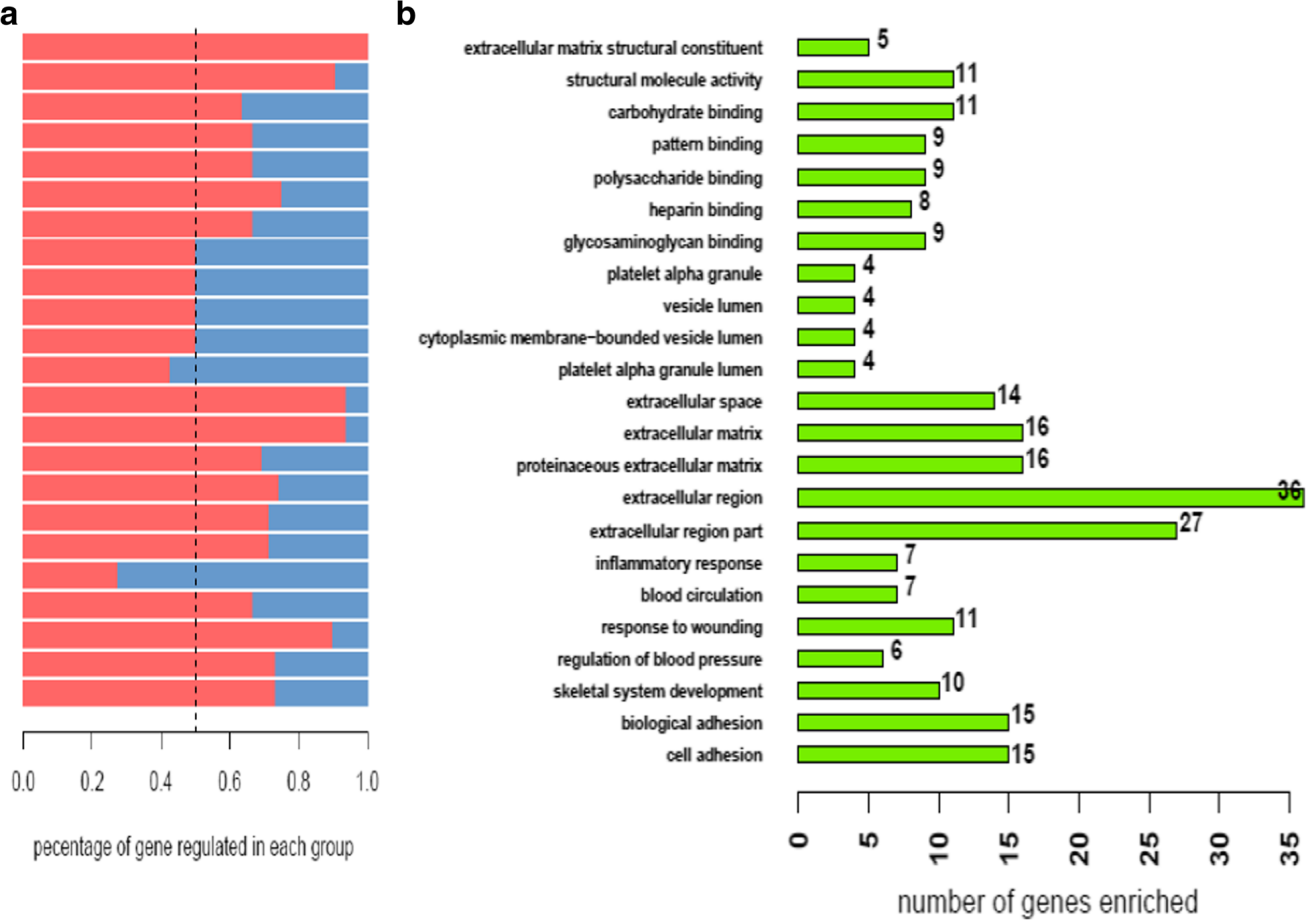
**a** Functional analysis in KEGG pathway (*P* < 0.05, ES > 1.5). **b** A diagram performed in order to find the number of collective genes. The size of the circle in the B is not related to the number of genes in the pathway or intersection

The results of functional annotation about KEGG pathways are shown in Fig. 2. COL1A1, COL1A2, COMP, THBS2, and THBS4, RELN participate in the pathways associated with ECM-receptor interaction and focal adhesion. COMP, THBS2 and THBS4 are significantly enriched in all three pathways.

### Topological analysis of PPI networks

**Fig. 3:**
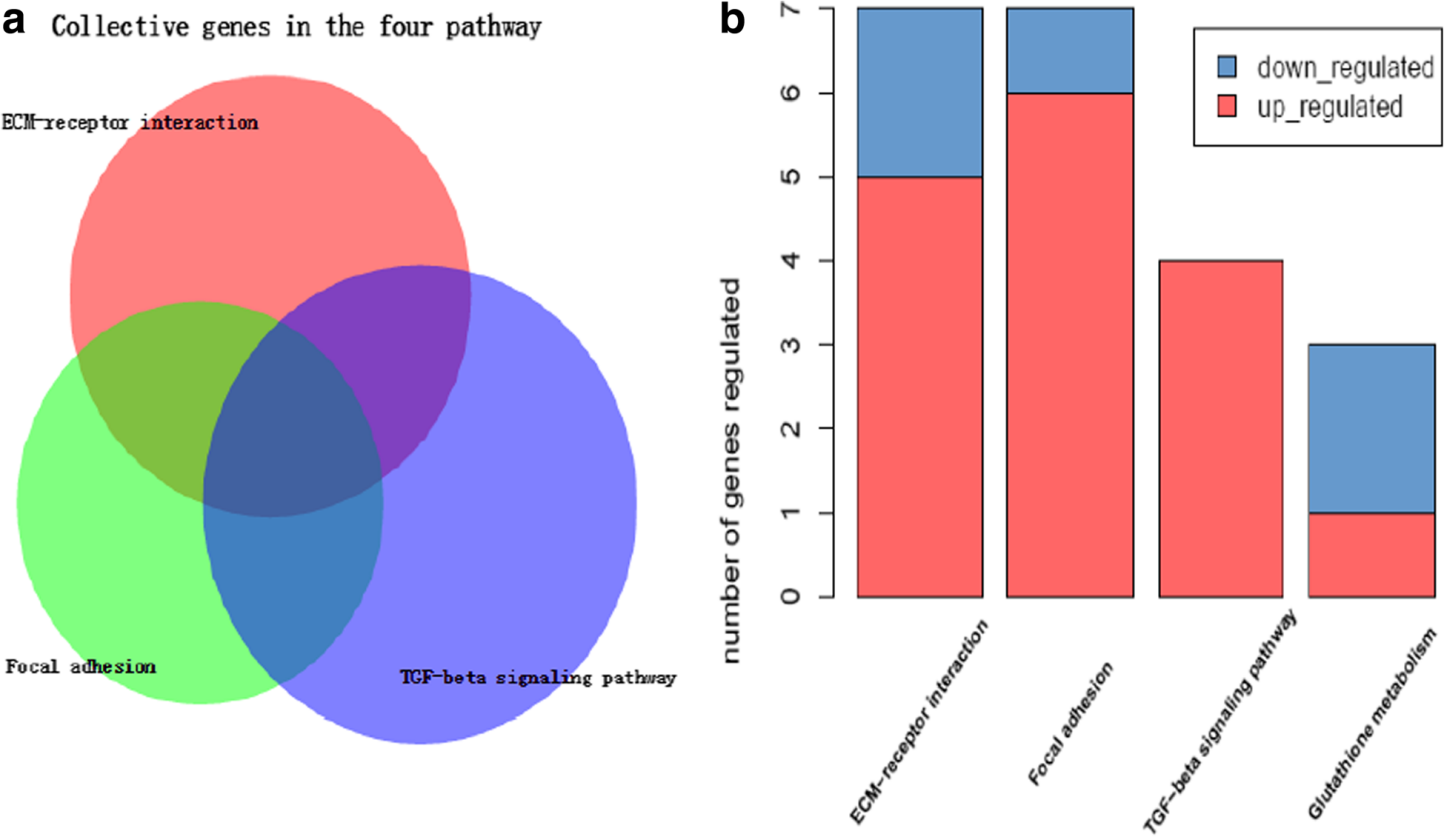
The protein interaction network constructed by STRING (**a**). 3 sub-networks (**b**, **c**, **d**) of PPI network performed using Cytoscape

**Fig. 4:**
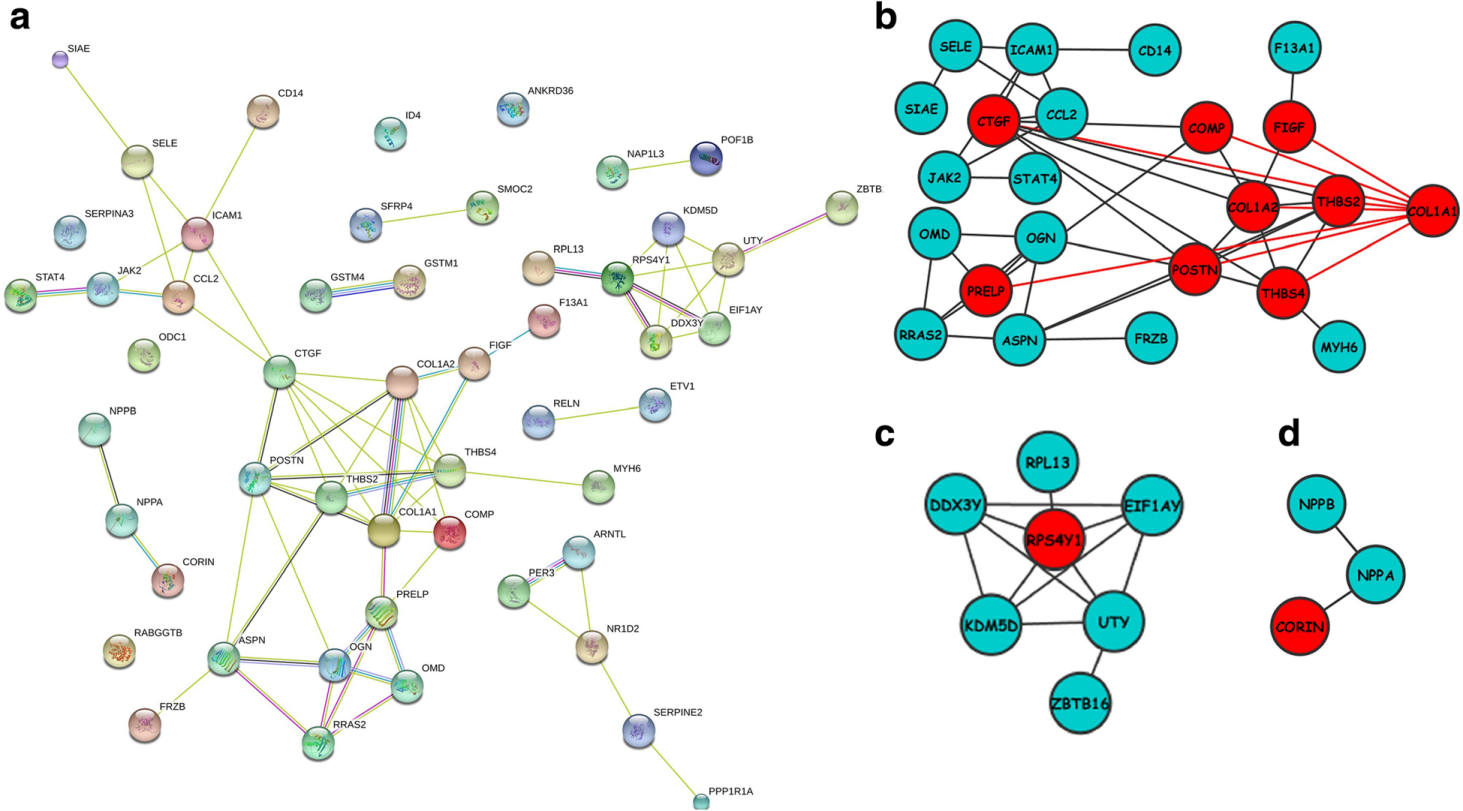
The expression level of selected 11 genes between control samples and DCM samples

Fifty-one significantly enriched gene were updated to STRING to construct the PPI network, and the PPI network was subsequently imported in Cytoscape to construct sub-networks. In sub-network 1, CTGF, COL1A1 (proteins with the highest degree of 8) and protein adjacent to them (COL1A2, THBS2, THBS4, POSTN, COMP, FIGF, PRELP) are highlighted. In sub-network 2 RPS4Y1 is in the central position. Sub-network three exhibit the three proteins with highest LogFC among all the sub-networks, as is all shown in Fig. 3. Table 1 shows fold changes of 11 DCMs and relevant *P* value, respectively. Figure 4 shows expression level of selected 11 genes between control and DCM samples. Among the 11 genes, nine of them are up-regulated while two are down-regulated.

**Table 1:** The expression level of 11 DEGs

Gene	Control Mean ± SD(log)	DCM Mean ± SD(log)	Log2 (Fold change)	*P*-value
collagen, type I, alpha 1(COL1A1)	9.46 ± 0.56	11.04 ± 0.72	1.50	0.001
collagen, type I, alpha 2(COL1A2)	8.73 ± 0.38	10.29 ± 1.00	1.50	0.004
cartilage oligomeric matrix protein(COMP)	6.50 ± 0.24	7.86 ± 0.68	1.32	5.18 × 10^-4^
connective tissue growth factor(CTGF)	8.51 ± 0.76	10.49 ± 0.52	1.93	1.50 × 10^-4^
periostin, osteoblast specific factor(POSTN)	10.09 ± 0.68	11.38 ± 0.89	1.48	0.007
thrombospondin 2(THBS2)	9.27 ± 0.25	10.39 ± 0.60	1.08	0.001
thrombospondin 4(THBS4)	10.53 ± 0.78	10.76 ± 0.73	1.47	3.13 × 10^-4^
corin, serine peptidase(CORIN)	8.57 ± 0.94	6.72 ± 1.15	−1.86	0.008
c-fos induced growth factor (vascular endothelial growth factor D)(FIGF)	8.78 ± 0.47	8.15 ± 0.53	−1.23	0.035
ribosomal protein S4, Y-linked 1(RPS4Y1)	8.10 ± 1.16	9.46 ± 1.90	1.38	0.039
proline/arginine-rich end leucine-rich repeat protein(PRELP)	9.71 ± 0.44	10.81 ± 0.55	1.10	0.002

### Analysis of transcription factor

**Fig. 5:**
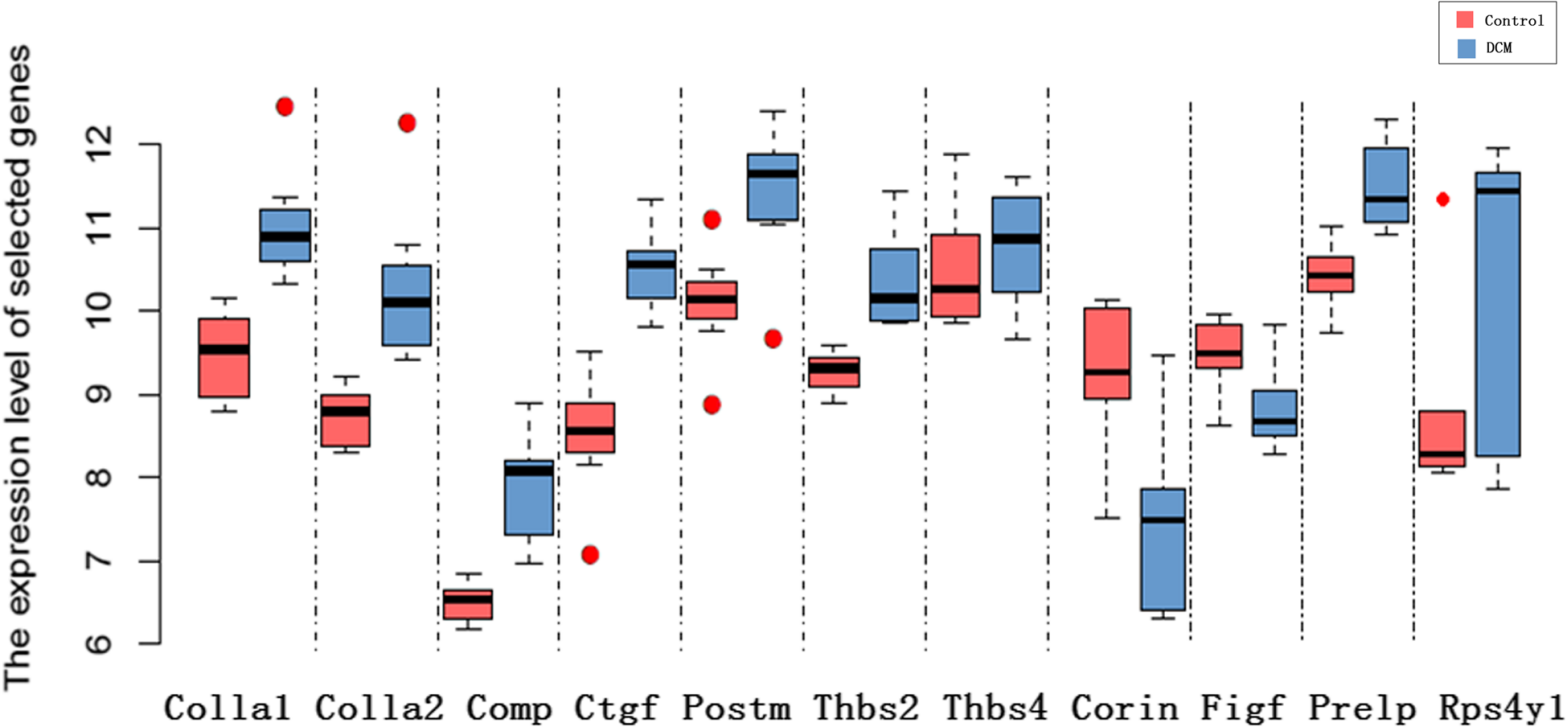
**a** Functional enrichment analysis based on Gene Ontology (*P* < 0.05, Benjamin< 0.01). **b** Number of differential expression genes enriched in each term

**Fig. 6:**
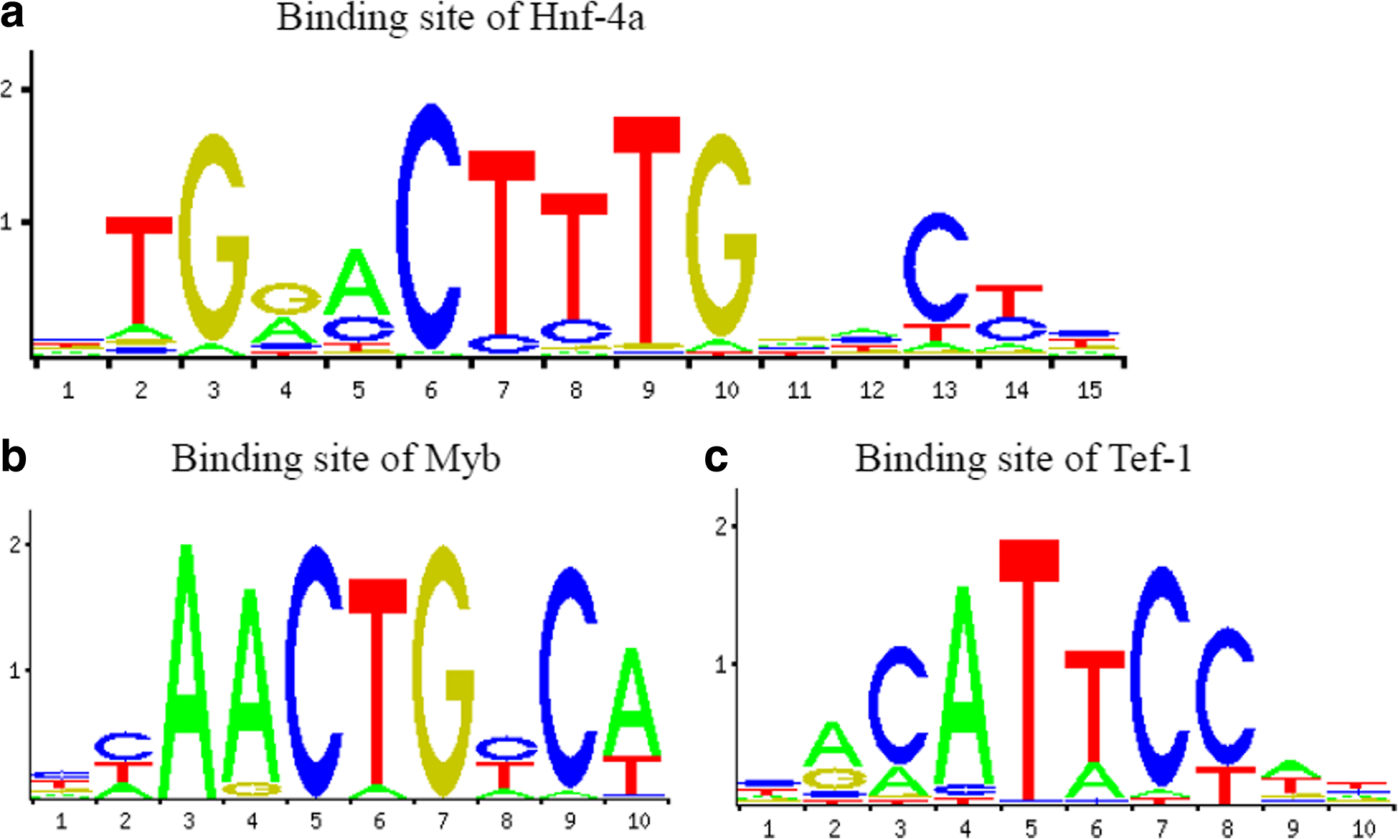
Transcription factors binding site predicted by JASPAR, Hnf-4a (Fig. 6a), Myb (Fig. 6b) and Tef-1 (Fig. 6c)

Transcription factor that modulate gene expression in DCM predicted by PASTAA is shown in Table 2. As shown in the Fig. 5a, Tef-1 and TFIIA transcription factor families play an important role in up-regulation of gene expression. TBP and TFIIA show features of constitutive expression as widely existing in transcription. By contrast, as shown in Fig. 5b, CORIN, FIGF of Myb families and Hnf-4 family are predicted to play an important role in down-regulation of gene expression. Figure 6 shows the transcription factor-binding site predicted by JASPAR.

**Table 2:** Results of PASTAA analysis

Rank	Matrix	Transcription Factor	Association Score	*P*-Value
a. TFs predicted to regulate up-regulated genes				
1	TATA_01	Tbp	4.51	2.22E-04
2	TEF1_Q6	Tef-1	4.1	5.01E-04
3	TFIIA_Q6	TfIIa-alpha/beta, TfIIa-gamma	3.252	2.81E-03
4	NFKAPPAB_01	Rela	2.876	6.51E-03
5	NRL_HAND	N/A	2.865	6.75E-03
6	GFI1_01	Gfi1	2.85	7.00E-03
7	ALPHACP1_01	N/A	2.718	9.92E-03
8	CREL_01	C-rel	2.587	1.15E-02
9	TAL1BETAITF2_01	Itf-2, Tal-1beta	2.553	1.22E-02
10	NFY_01	N/A	2.509	1.44E-02
b. TFs predicted to regulate down-regulated genes				
1	VMYB_01	V-myb	3.123	6.94E-04
2	HNF4ALPHA_Q6	Hnf-4, Hnf-4alpha	3.043	9.08E-04
3	MYB_Q3	C-myb	2.646	4.16E-03
4	MYB_Q5_01	B-myb, C-myb	2.646	4.16E-03
5	CDC5_01	Cdc5	2.425	6.93E-03
6	CEBP_Q2	C/ebp, C/ebpalpha	2.425	6.93E-03
7	AP1_Q4_01	Fosb, Fra-1	2.123	1.23E-02
8	ZTA_Q2	N/A	2.123	1.23E-02
9	AP1_Q2_01	Fosb, Fra-1	2.044	1.23E-02
10	COUP_01	Coup-tf1, Hnf-4alpha1	2.044	1.23E-02

### Acquisition of target genes of drugs

Gene set A and B was uploaded to Connectivity Map to search for potential drugs, Table 3 shows predicted drug molecules that may induce/inhibit DCM-associated gene expression. Predicted molecule in Table 2 was uploaded to NCBI PubChem database to search for target genes of receptors. 19 target genes are significantly correlated with DCM, as shown in Table 4. Numbers in the brackets stand for the count of drugs. CTGF, POSTN, CORIN, FIGH have higher amount of drugs. Collagen family and THBS2, THBS4 are also ranked highly.

**Table 3:** Chemical compounds identified by Connectivity map and list the top 20 ranked by P-value

rank	cmap name	Mean	n	enrichment	*P*-value
1	tridihexethyl	−0.64	4	− 0.883	0.00044
2	Prestwick-682	−0.509	4	−0.848	0.00097
3	fluoxetine	−0.494	4	−0.806	0.00271
4	pirlindole	−0.668	3	−0.884	0.00312
5	methyldopa	0.382	5	0.732	0.00318
6	ciclacillin	0.494	4	0.789	0.00391
7	sulfadiazine	0.565	5	0.717	0.00409
8	khellin	−0.418	5	−0.708	0.00467
9	PNU-0251126	−0.358	6	−0.656	0.00467
10	metampicillin	0.389	5	0.704	0.00537
11	CP-645525-01	−0.558	3	−0.861	0.00553
12	minoxidil	−0.446	5	−0.694	0.00597
13	spiradoline	−0.478	4	−0.756	0.00718
14	PHA-00816795	−0.719	2	−0.942	0.00722
15	acenocoumarol	0.509	5	0.687	0.00741
16	cefotaxime	0.561	5	0.682	0.00845
17	metrizamide	0.487	4	0.731	0.01044
18	Y-27632	−0.674	2	−0.922	0.01223
19	bacampicillin	−0.354	4	−0.718	0.01271
20	levocabastine	0.274	4	0.718	0.01297

Mean:the arithmetic mean of the connectivity scores for corresponding instances, N:The number of instances, Enrichment: The degree of enrichment of the given instance in all instances, P-value:an estimate of the likelihood that the enrichment of a set of predicted potential drugs of all CMap drugs would be observed by chance

**Table 4:** The genes targeted by the 20 potential drugs from CMap

CTGF (14) UP	POSTN (11) UP	COMP (8) UP	COL1A1 (8) UP	COL1A2 (8) UP
THBS2 (8) UP	THBS4 (8) UP	PRELP (7) UP	STAT4 (3) UP	FRZB (3) UP
RPS4Y1 (3) UP	ID4 (2) UP	ASPN (2) UP	SSPN (2) UP	
CORIN (7) DOWN	FIGF (5) DOWN	FCN3 (3) DOWN	CCL2 (2) DOWN	CD14 (1) DOWN

The number in the parentheses represent the number of associated CMap drugs, UP and DOWN represent up or down-regulated of corresponding gene

## Discussion

Microarray analysis, an optimal approach to identify differentially expressed gene, helps to define an early diagnosis and lower misdiagnosis rate [21]. So far, the application of microarray analysis has revealed considerable bioprocesses associated with DCM. Barth et al [5] predicted and constructed a gene set including 27 genes differentially expressed, among which 25 were later confirmed. Micaela et al [22] analyzed ion channel-associated gene differential expression. They reported that ion channel-associated gene such as SCN2B. KCNJ5, CLIC2 play an important role in related bioprocesses. The present study aims at underlining the effects of regulation by transcription factors on differential gene expression. Effectiveness of target gene set is confirmed by referring to existing database.

By comparing differentially expressed genes in DCM samples with that from healthy controls, we predict that CORIN, FIGF, CTGF, COL1A1, COL1A2, THBS4, THBS4, POSTN, COMP, PRELP and RPS4Y1 may play a role in development of DCM. By the way, CORIN (encoding production could convert pro-ANP to bioactive ANP) is one of the known marker genes, the purpose of this study is to explore the mechanism underlying the differential expression level.

Cardiac remodeling characterized by collagen deposition in extracellular matrix [23] and myocardial fibrosis leading to heart failure [24, 25]. Expression level changes of genes encoding proteins associated with: 1) ECM-receptor interaction, 2) focal adhesion, 3) TGF-beta signaling pathway may play an integral role in development of systolic dysfunction. Up-regulation of CTGF in TGF-beta signaling pathway can lead to an increased deposition of type 3 collagen (COL3A1) and type 1 collagen (COL1A1, COL1A2 and COL1A3) [26, 27]. Furthermore, excessive collagen may cause myocardial fibrosis and heart failure. THBS2 and THBS4, existing in all three pathways that encode fibronectin are also modulated by CTGF. Previous study shows that up-regulation of THBS1 and THBS4 may lead to matri-cellular protein deposition, and subsequently resulting in DCM, heart failure or death. FIGF (also known as vascular endothelial growth factor-D; VEGF-D) in ECM-receptor interaction may also participate in the pathology of DCM. Gong X et al. [28] reported that VEGF preserve cardiac function after intra-myocardial transplantation in a DCM mouse by reducing cellular apoptosis and myocardial fibrosis in addition to enhanced angiogenesis, indicating that down-regulation of FIGF may improve cardiac function and preserve myocardial cells.

Differential expression gene exhibit up-regulation in GO terms of extracellular matrix and focal adhesion [29]. Mal-regulation of extracellular matrix is associated with progression of cardiac remodeling and heart failure. COMP encodes a non-collagen protein in ECM and previous studies have demonstrated that abnormal expression of COMP result in myocardial cell apoptosis and loss of myofilament [30]. COMP, COL1A1, COL1A2 and PRELP are up-regulated in ECM-receptor interaction and focal adhesion, inducing a component change in extracellular matrix [31].

POSTN bond with heparin, and is showed in the PPI network co-expressed with COL1A1, COL1A2, THBS4 and CTGF. High impression level of POSTN in myocardial fibroblast contributes to cardiac remodeling [32]. Therefore, highly co-expression of both POSTN and CTGF (regulate heparin in fibroblasts) may be associated with antagonism. However, it is unknown whether POSTN is regulated by CTGF. The co-expression of RPS4Y1, DDX3Y, EIF1AY and RPL13 were discovered. Heidecker et al. [33] demonstrated that they are associated with idiopathic DCM, but the expression level differentiates significantly and the mechanism remains unclear.

Transcription factors may affect the differential expression genes in DCM. Tef-1 (also known as TEAD1, TCF-13), a transcription enhancer, promotes the expression of troponin, myosin and actin [34, 35]. TFIIA, a member of TF family, exists ubiquitously in global tissues, participating in the formation of transcription initiation complex as an intra-nuclear protein [36]. It may trigger the up-regulation of differentially expressed genes. MYB [37], known as a transcription activator, encodes transcription factors of myb family. MYB has 1) a N-terminal DNA binding domain, 2) a transcription activating domain in the center [38] 3) a C-terminal domain associated with inhibition of transcription. The C-domain may be responsible for down-regulation of FIGF and CORIN. Hnf-4 transcription factor family is mainly expressed in kidney and liver [39]. Given that it is a transcription activator, the inhibition may down-regulate the expression of CORIN, FIGF and subsequentially the down-regulation of ANP level.

Re-localization of drugs is a cost-effective approach so that the development of CMap is engaged in efficiently predicting potential targets that drugs can aim at. Tridihexethyl, fluoxetine and pirlindole can inactivate G-protein-coupled receptor. Fluoxetine, methyldopa and sulfadiazine can inactivate cystic fibrosis trans-membrane conductance regulator and sequentially down-regulate the expression of Collagen, THBS, COMP and PRELP. Methyldopa, sulfadiazine and khellin may further regulate the gene expression by regulating the translation. Besides, up-regulated CTGF, POSTN and down-regulated CORN, FIGF are seen as the major targets in CMap prediction, indicating that combination of multiple drugs may achieve a better treatment.

However, up or down regulation of specific genes may not be responsible for pathogenesis of the relevant diseases [40, 41]. They may be only a downstream reaction or byproducts, such as s NPPB(logFC> 4.2)or NPPA(logFC> 2.5)in DCM. Age, gender, multi-drug therapy, etiology and individual variation may also cause differential gene expression. That is the limitation of the present study. In addition, we all know now that myofibril gene (protein) mutations are associated with the development of various cardiomyopathies including DCM. It is necessary in the future to carry out more analyses including the above-mentioned factors and myofibril protein mutations. Besides, differentially expressed genes in the same expression profile vary among different filters. These factors have also been taken into consideration in drawing any conclusions.

## Conclusions

Bioinformatics-based analyses reveal the targeted genes probably associated with cardiomyopathy, which provide clues for pharmacological therapies aiming at the targets. Further studies considering other factors such as age, gender, multi-drug therapy, etiology and individual variation should be carried out in the future and bench work experiments should be performed to verify the relationship between the DEGs and the development of DCM.

## Abbreviations

BMCs: Bone marrow-derived cells
BNP: B-type natriuretic peptides
CMap: Connectivity map
DAVID: Visualization and integrated discovery
DCM: Dilated cardiomyopathy
DEGS: Differential expression of genes
ECM: Extracellular matrix
G-CSF: Granulocyte colony-stimulating factor
GEO: Expression Omnibus Gene
GO: Gene ontology
KEGG: The Kyoto Encyclopedia of Genes and Genomes
PCR: Polymerase chain reaction
PPI: Protein-protein interaction network
STRING: Search tool for the retrieval interacting genes
TGF-β: Transforming growth factor beta
